# Estradiol and non-REM sleep attenuate physiological and emotional responses to social-evaluative stress in healthy women

**DOI:** 10.1186/s12916-025-04508-x

**Published:** 2025-12-01

**Authors:** Elisa M. S. Meth, Diana A. Nôga, Frank Wedzinga, Abdullah Almajni, André P. Pacheco, Viviana Rossi, Per C. Simonsson, Céline Heel, Camilla Zetterlund, Samira F. M. Noory, Michaela Danek, Pei Xue, Christian Benedict

**Affiliations:** 1 Department of Pharmaceutical Biosciences, Uppsala University, Husargatan 3, Box 593, Uppsala, 751 24 Sweden; 2 Department of Research and Innovation, Division of Mental Health and Addiction, Oslo University Hospital, Sognsvannsveien 21, Oslo, 0372 Norway; 3 Institute of Clinical Medicine, Faculty of Medicine, University of Oslo, Postboks 1039 Blindern, Oslo, 0315 Norway

**Keywords:** Estradiol, Non-REM sleep, Social stress, Emotion regulation, Autonomic arousal, Cognitive appraisal

## Abstract

**Background:**

This study examined whether individual differences in estradiol and sleep predict autonomic and cognitive responses to social-evaluative stress in healthy young women.

**Methods:**

Forty-two healthy women underwent overnight in-laboratory sleep monitoring, followed by a social-evaluative stress task the next morning, which involved listening to a playback of their own karaoke singing. Pre-task estradiol levels were measured via blood sampling. Autonomic responses were assessed using pupil dilation during the playback, while cognitive responses were indexed by subjective stress ratings collected immediately afterward.

**Results:**

Higher estradiol concentrations (range: 40–1129 pmol/L) were associated with reduced peak pupil dilation (range: 0.21–1.70 mm above the 95% confidence interval upper bound of baseline) and cumulative pupil dilation (total pupil expansion exceeding the individual baseline over 45 s; range: 64–3139 mm, *p* < 0.001), indicating lower autonomic arousal. In contrast, estradiol was not associated with subjective stress ratings (range: 10–98 mm on a 100-mm scale). Greater N3 sleep duration (range: 27–172 min), but not N1 or N2, was associated with lower subjective stress (*p* = 0.032), whereas REM sleep duration (range: 53–152 min) showed no association with either outcome. None of the sleep measures was significantly related to pupillometry outcomes, and no interaction effects were observed between estradiol and sleep measures.

**Conclusions:**

Estradiol and N3 sleep appear to independently modulate distinct facets of the response to social-evaluative stress: higher estradiol levels were associated with reduced autonomic arousal, while greater N3 sleep duration was linked to lower subjective stress. By revealing parallel pathways through which estradiol and sleep shape physiological and cognitive reactions to stress, this work contributes to a more nuanced understanding of the biological factors that may influence mental health and stress resilience.

## Background

Modern life in a constantly connected, 24/7 society exposes individuals to frequent and often chronic psychological stress [1]. Women, in particular, face a dual burden of full-time employment and primary caregiving responsibilities, contributing to disproportionately higher rates of depression, anxiety, and burnout [2, 3].

To understand how individuals cope with such ongoing challenges, researchers have increasingly turned to the concept of resilience, the ability to maintain or recover psychological and physiological stability in the face of stress. Resilience encompasses both subjective elements, such as perceived coping capacity [4], and objective indicators, including physiological responses governed by the autonomic nervous system (ANS), particularly the sympathetic nervous system (SNS), which mobilizes the body in response to threat or challenge [5]. Arousal, in this context, refers to the body’s immediate physiological reaction to a stressor, involving heightened SNS activity and measurable outputs like increased heart rate, blood pressure, or pupil dilation.

Sleep has emerged as a critical regulator of stress reactivity and emotional resilience. Both the quantity and the architecture of sleep influence how individuals respond to and recover from emotional challenges and stressors [6–9]. In particular, rapid eye movement (REM) sleep plays a key role in emotional regulation [10]. Reduced REM sleep amplifies amygdala reactivity to negative stimuli and disrupts its communication with prefrontal regions responsible for top-down regulation [11]. Sleep deprivation also heightens sensitivity to negative social cues, a pattern that can be reversed with naps containing REM sleep [12], underscoring the restorative role of REM in emotion regulation. Recent findings suggest that non-REM stage 3 (N3) sleep, the deepest stage of sleep, also plays a vital role in next-day emotional stability and autonomic stress reactivity. Greater N3 sleep has been associated with lower anxiety [13] and preserved positive affect [14, 15], while experimental suppression of N3 sleep in a sample of men and women increases autonomic reactivity to social-evaluative stress [16].

Another biological factor implicated in modulating stress responses is estradiol, a key ovarian hormone that fluctuates across the menstrual cycle. In naturally cycling women, higher estradiol levels have been linked to enhanced functional connectivity between prefrontal and limbic brain regions involved in emotion regulation [17]. Nevertheless, findings are mixed: some studies report that estradiol heightens stress reactivity [18, 19], while others suggest it has a buffering effect, reducing stress-related markers such as blood pressure and catecholamine levels [20]. Evidence from animal models further illustrates this complexity. Estradiol, or selective activation of estrogen receptors (ERs), can influence hypothalamo-pituitary-adrenal axis responses to stress in a receptor-dependent manner: ERα activation generally enhances adrenocorticotropic hormone and corticosterone responses, whereas ERβ activation suppresses them, underscoring receptor-specific pathways through which estradiol shapes stress reactivity [21]. For example, Smiley et al. [22] utilized a social stress model, termed witness stress in naturally cycling female rats and found that repeated exposure induced anxiety- and depression-like behaviors, as well as hypervigilance. These effects were accompanied by increased expression of ERβ and corticotropin-releasing factor in the central amygdala. Notably, pharmacological inhibition of ERβ in this region attenuated both behavioral and neuronal consequences of stress, pinpointing to a critical role of ERβ signaling in modulating stress reactivity [22]. The inconsistencies from human studies, complemented by mechanistic evidence from animal models, underscore the importance of studying estradiol under natural hormonal conditions, and in tandem with other resilience-promoting factors like sleep.

In the present study, we investigated whether individual differences in prior-night sleep and morning serum estradiol levels predicted autonomic and subjective responses to a psychosocial stressor. We tested 42 healthy, young, naturally cycling women. As a standardized social-evaluative stressor, participants, without prior warning, listened to an audio recording of their own karaoke performance while seated in a room with a silent, non-responsive experimenter present. This setup was designed to elicit social-evaluative stress, as it combines self-exposure with perceived external judgment in the absence of feedback. Autonomic arousal was indexed using pupillometry, a noninvasive method for tracking pupil dilation. Pupil size is a rapid, involuntary, and precise indicator of ANS activity. It increases with sympathetic activation through noradrenergic signaling from the locus coeruleus [23, 24] and constricts via parasympathetic cholinergic input from the Edinger-Westphal nucleus [25]. Because pupil dilation occurs within milliseconds in response to stress [26], pupillometry provides a sensitive and noninvasive window into unconscious, stress-related arousal. Subjective stress appraisal, which is shaped by cognitive evaluation of the stressor, was assessed immediately after the task using a visual analog scale (VAS).

We hypothesized that greater amounts of REM and non-REM (particularly N3) sleep on the night prior to testing, along with higher morning estradiol concentrations, would be associated with reduced pupil dilation and lower subjective stress. Additionally, we explored whether interactions between sleep and estradiol levels might influence these stress responses.

## Methods

### Study cohort

A total of 673 women expressed interest in participating in the study. Of these, 288 completed an online screening questionnaire to determine eligibility. Exclusion criteria included age below 18; a self-reported history of physical, psychiatric, or sleep-related disorders; habitual sleep onset before 22:00 or after 00:00; self-reported bad sleep quality or recurrent insomnia symptoms; employment involving occasional or permanent night shifts; use of hormonal contraceptives (participants were required to have discontinued hormonal contraceptives at least 3 months prior to enrollment to minimize potential residual effects on hormonal regulation and study outcomes) or other medications; more than five standard units of alcohol or caffeine beverages per day; drugs or nicotine usage; irregular menstrual cycles or cycle lengths outside the range of 26–35 days; premenstrual disorders; planned travel across time zones during the study period; and presence of obstructive sleep apnea, defined as five or more apneas or hypopneas per hour as measured by a Withings Sleep Analyzer during the adaptation night [27].

Participants who met all inclusion criteria (*n* = 60) were invited to take part in an adaptation night at the sleep laboratory in the Department of Pharmaceutical Biosciences, Uppsala University, Sweden. Sleep was measured using the Dreem headband, a wearable reduced-montage electroencephalography (EEG) device with five dry electrodes (O1, O2, FpZ, F7, F8), a 3D accelerometer, and a pulse oximeter, which together automatically score sleep stages. The device has been validated against polysomnography, showing high agreement for EEG signals and sleep staging with 83% accuracy compared to expert scorers [28]. Of the 60 eligible participants, two withdrew before the adaptation night, one was excluded due to obstructive sleep apnea, and an additional three chose not to continue with the study after completing the adaptation night. Additional exclusions were made due to missing blood samples (e.g., participants withdrawing consent) and unavailable eye-tracking data (e.g., equipment maintenance or calibration failures). After these exclusions, the final sample comprised 42 healthy, naturally cycling young women (age range: 21–33 years) who were included in the final analyses.

All study procedures were conducted in accordance with the Declaration of Helsinki and approved by the Regional Ethical Review Board in Uppsala, Sweden (DNR 66–2021/3.1). Written informed consent was obtained from all participants prior to participation. Participants received monetary compensation. Regional Ethical Review Board in Uppsala, Sweden, did not classify the study as a clinical trial. It was nonetheless registered on ClinicalTrials.gov (NCT06683248). The analyses presented here form part of a larger investigation into the neurological, metabolic, and immune consequences of sleep and sleep deprivation.

### Experimental scheme

**Fig. 1:**
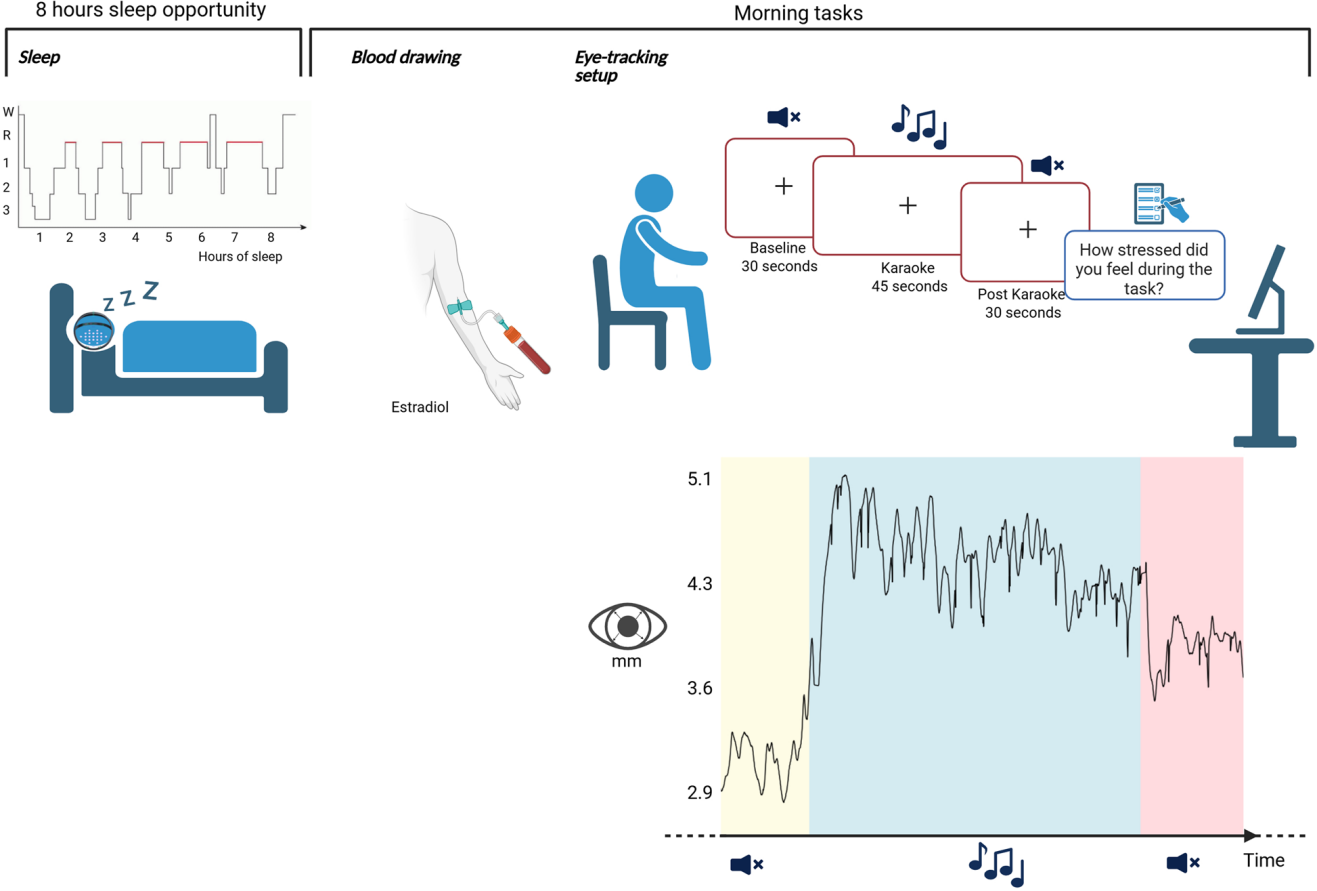
Overview of study protocol. Forty-two women of reproductive age who were naturally cycling (i.e., not using hormonal contraceptives) participated in the study. Each underwent an 8-h in-laboratory sleep opportunity (23:00–07:00), during which a headband was used to assess sleep. The following morning, venous blood samples were collected approximately 30 min after awakening to quantify estradiol concentrations. Participants then completed an eye-tracking task comprising three phases: a 30-s resting baseline, 45 s of karaoke playback (used as a social-evaluative stressor), and a 30-s post-task period. Pupil dilation was continuously recorded throughout the task. Immediately following the playback, participants rated their perceived stress using a visual analog scale (VAS). For illustrative purposes, the pupil dilation response of one randomly selected participant across the task timeline is shown

As illustrated in Fig. 1, participants were provided with an 8-h sleep opportunity during an in-laboratory overnight session (lights off at 23:00; lights on at 07:00). Sleep was recorded using the Dreem headband. Approximately 30 min after awakening, venous blood samples were collected using Gold Top SST (Serum Separator Tubes), which contain a clot activator and a polymer gel for serum separation. Concentrations of estradiol, progesterone, follicle-stimulating hormone (FSH), and luteinizing hormone (LH) were analyzed via electrochemiluminescence immunoassay (Roche Cobas Pro, Roche Diagnostics). Four hours post-awakening, participants completed the karaoke eye-tracking task (see below for details). This timing was chosen to avoid overlap with hormonal fluctuations typically occurring as part of the sleep–wake transition, such as the cortisol awakening response [29]. Participants also reported the day of their last menstruation (defined as day 1 of the cycle) and their average cycle length.

### Singing paradigm and stress measurement

During the adaptation night, participants performed a karaoke rendition of *Chandelier* by Sia (Furler & Shatkin, 2014; 1000 Forms of Fear, Monkey Puzzle Records/RCA). This song was selected because pilot experiments with female students indicated that it was perceived as vocally demanding. At the time of recording, the melody was presented to participants via headphones (Sony WH-1000XM4, Sony Corporation, Tokyo, Japan), while the lyrics were displayed on a screen, with a moving dot indicating the current position to guide timing. Participants’ singing was recorded using a professional microphone system (NEEWER USB Microphone 4-in-1 Kit for Windows and Mac, Shenzhen, Guangdong, China) fixed on a holder. Participants were not informed that their performance would later be used.

To enhance the social-evaluative pressure during the recording, an experimenter remained in the room throughout the task, providing a silent but observable presence. The present design aligns partly with other singing paradigms, the short Sing-a-Song Test [30]. For the playback task, a 45-s segment was extracted from each participant’s recording, beginning with the final lyrics “*1, 2, 3, 1, 2, 3, drink*” at the first pre-chorus and ending with the second last lyrics at the first chorus (“*I’m gonna swing from the chandelier*”).

### Eye tracking procedure and paradigm

During the karaoke replay, scheduled for the morning after the overnight sleep session, participants were seated 50–70 cm from the eye tracker monitor, with their head stabilized using a chin rest to minimize movement. To properly capture their pupil response, participants were instructed to fixate on a centrally presented crosshair, remain still, and refrain from speaking or laughing throughout the task. During the playback sequence, which was played aloud in the room, participants experienced three consecutive blocks: a 30-s silent baseline, a 45-s playback of their own recorded singing (karaoke), and a 30-s post-stimulus silent period (post-karaoke). An experimenter remained in the room to enhance the social-evaluative nature of the situation, though they stayed silent to avoid introducing additional social cues. Playback volume was standardized at 70 dB SPL, and each participant heard the same 45-s segment from their own recording to ensure consistency in auditory exposure. Immediately following the karaoke replay, participants rated their subjective stress levels during the karaoke task using a 100-mm VAS, ranging from “not at all stressed” to “extremely stressed.” These ratings offered a direct window into participants’ high-level cognitive appraisals of stress.

### Pupillometry data acquisition and preprocessing

Participants’ visual gaze was recorded using a Tobii Pro Spectrum eye tracker (Tobii© Technology AB, Stockholm, Sweden), operating at 60 Hz with binocular tracking. Visual stimuli were presented on a 23.8-inch monitor (1920 × 1080 resolution) against a uniform gray background with a luminance of approximately 23.24 cd/m^2^. A centrally located fixation cross was used to direct attention. The ambient lighting in the testing environment was maintained at approximately 180 lx at eye level.

To analyze the pupil size data, procedures outlined elsewhere [31] and the guidelines in the PupillometryR package [32] were followed, using Python (version 3.13.3) for preprocessing. The process began with a deblinking procedure, removing pupil data during periods marked as “Eyes Not Found” by the eye tracker—typically indicative of blinks or signal loss—as well as the six samples immediately before and after each such period (± 100 ms at 60 Hz), to reduce artifacts caused by eyelid movements. Next, extreme pupil diameter values—those deviating more than three standard deviations from the median—were discarded to minimize the influence of outliers. To further refine the signal, a smoothing technique was applied using a Hann window of size 5, which attenuates high-frequency noise while preserving natural changes in pupil size over time. Short gaps in data (up to 750 ms or approximately 45 samples) were interpolated linearly, whereas longer gaps remained unfilled to avoid artificially inflating pupil responses. These preprocessing steps were applied separately for each task segment (baseline, karaoke, post-karaoke), maintaining the temporal integrity of each condition. For the baseline segment, only the last 20 s were analyzed to ensure the pupil had stabilized. The first 10 s were excluded to allow for this stabilization. The karaoke and post-karaoke segments were analyzed entirely.

To measure pupil dilation in response to the karaoke playback, we first calculated the upper bound of the 95% confidence interval (95% CI) of the pupil size during the last 20 s of the baseline period for each participant. This upper bound served as a threshold for detecting pupil dilation during both the karaoke and post-karaoke periods. Peak pupil dilation was calculated as the maximum pupil size during the karaoke period, minus the upper baseline threshold. For example, if a participant’s maximum pupil size during karaoke was 5.0 mm and the baseline threshold was 3.0 mm, the baseline-adjusted peak dilation would be 2.0 mm. Cumulative pupil dilation was calculated by summing all the positive deviations (i.e., all moments where pupil size exceeded the baseline threshold) during the karaoke period. Only data points above the 95% CI upper bound were included in this calculation. Finally, to simplify the analysis, we averaged the pupil size data from both the left and right eyes at each time point, providing a single continuous measurement per participant.

### Statistical analyses

All statistical analyses were performed using IBM SPSS Statistics, version 26 (IBM Corp., Armonk, NY, USA). Generalized linear models (GzLMs) were used to assess the impact of sleep and hormonal factors on stress-related outcomes. Unless otherwise noted, all GzLMs specified a normal distribution with an identity link function and included the following continuous between-subject predictors: morning blood estradiol concentration, time spent in non-REM sleep, and time spent in REM sleep. To account for individual differences in stress-coping abilities, we included an emotional stability score (the inverse of neuroticism), derived from the Big Five Inventory ( [33]; filled out during the adaptation night session). We also investigated possible interaction effects between estradiol and time spent in non-REM or REM sleep by including their interaction terms along with the corresponding main components in separate GzLMs.

The primary outcome variables were peak pupil diameter during the karaoke playback; cumulative pupil dilation during the karaoke playback; and subjective stress ratings collected immediately after the cessation of the eye-tracking paradigm. Unless otherwise specified, results are presented as estimated marginal means with corresponding 95% CIs. Bivariate correlational analyses were conducted using either Pearson’s or Spearman’s correlation coefficients, depending on the normality of the variables’ distribution (assessed using the Kolmogorov–Smirnov test). Finally, we conducted several sensitivity analyses. In one analysis, we examined whether the effects of estradiol on pupil dilation and subjective stress ratings were influenced by progesterone. In a separate analysis, we tested whether our main findings held when participants’ neuroticism scores were removed from the models, given that this trait may influence predictors such as sleep and estradiol. In a third sensitivity analysis, we re-ran our models using total sleep time (TST) as the predictor instead of REM and non-REM durations to assess whether TST explained more variance in the outcomes than individual sleep stages. Across all analyses, a *p* value of less than 0.05 was considered statistically significant.

## Results

### Descriptive

A total of 42 women (mean age = 24.8 years, SD = 2.8) participated in the study. Scores on the emotional stability scale ranged from 10 to 44 points, with a mean score of 31.0 points (SD = 7.4). During the in-laboratory sleep night, participants obtained an average of 432 min of total sleep (SD = 34, range = 348–474 min), consisting of 330 min of non-REM sleep (SD = 25, range = 271–371 min), 102 min of REM sleep (SD = 24, range = 53–152 min), and 24 min of wake after sleep onset (SD = 21, range = 6–82 min). Morning blood estradiol concentrations, measured approximately 30 min after awakening, averaged 361 pmol/L (SD = 272, range = 40–1129 pmol/L). Menstrual cycle phase was determined according to Swedish clinical references [34], based on hormone levels (estradiol, progesterone, FSH, LH) and supplemented with self-reported data (last menstruation and cycle length), in line with previous recommendations [35]. Of the 42 participants, 27 (64.3%) were in the follicular phase, 13 (31.0%) in the luteal phase, and 2 (4.8%) near ovulation.

### Pupil dilation response to playback of own karaoke singing

**Fig. 2:**
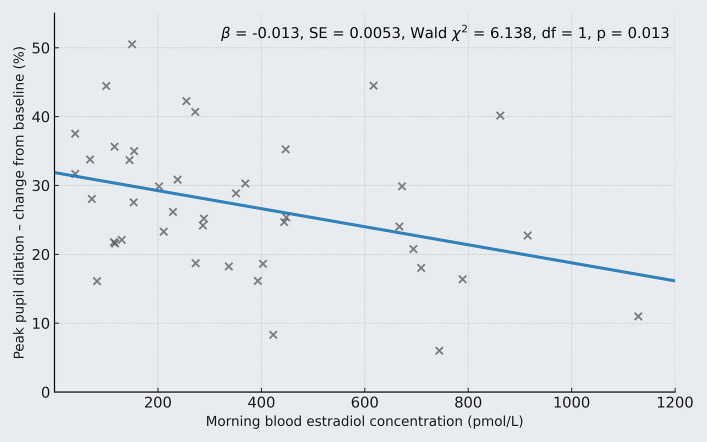
Elevated morning estradiol concentrations were significantly associated with reduced peak pupil dilation during the karaoke replay. The blue line represents the predicted association, and the estimates shown are derived from a generalized linear model, with morning blood estradiol concentrations measured after sleep entered as a continuous predictor and peak pupil response (expressed as a percentage from baseline) as the outcome variable. Each gray “x” represents an individual participant’s observed data point. For adjusted estimates of baseline-corrected peak pupil diameter, see the Results section

The mean baseline pupil diameter in the studied cohort was 3.499 mm (range: 2.687 to 5.654 mm), with the upper bound of the 95% CI ranging from 2.700 to 5.666 mm among women. During the karaoke replay, the mean increase in peak pupil dilation from each individual’s upper baseline bound was + 0.94 mm (range: + 0.21 to + 1.70 mm). GzLMs results indicated that women with higher blood concentrations of estradiol exhibited smaller peak pupil diameters during the karaoke playback compared to those with lower estradiol levels (*B* = − 0.001 mm per change in pmol/L estradiol, SE = 0.0002, Wald χ^2^ = 14.257, df = 1, *p* < 0.001). In other words, based on model estimates, a 500 pmol/L difference in blood estradiol concentration between two women would correspond to an estimated difference of approximately 0.5 mm in peak pupil diameter. The percentage change in peak pupil diameter relative to the upper 95% CI of baseline values is shown in Fig. 2.

**Fig. 3:**
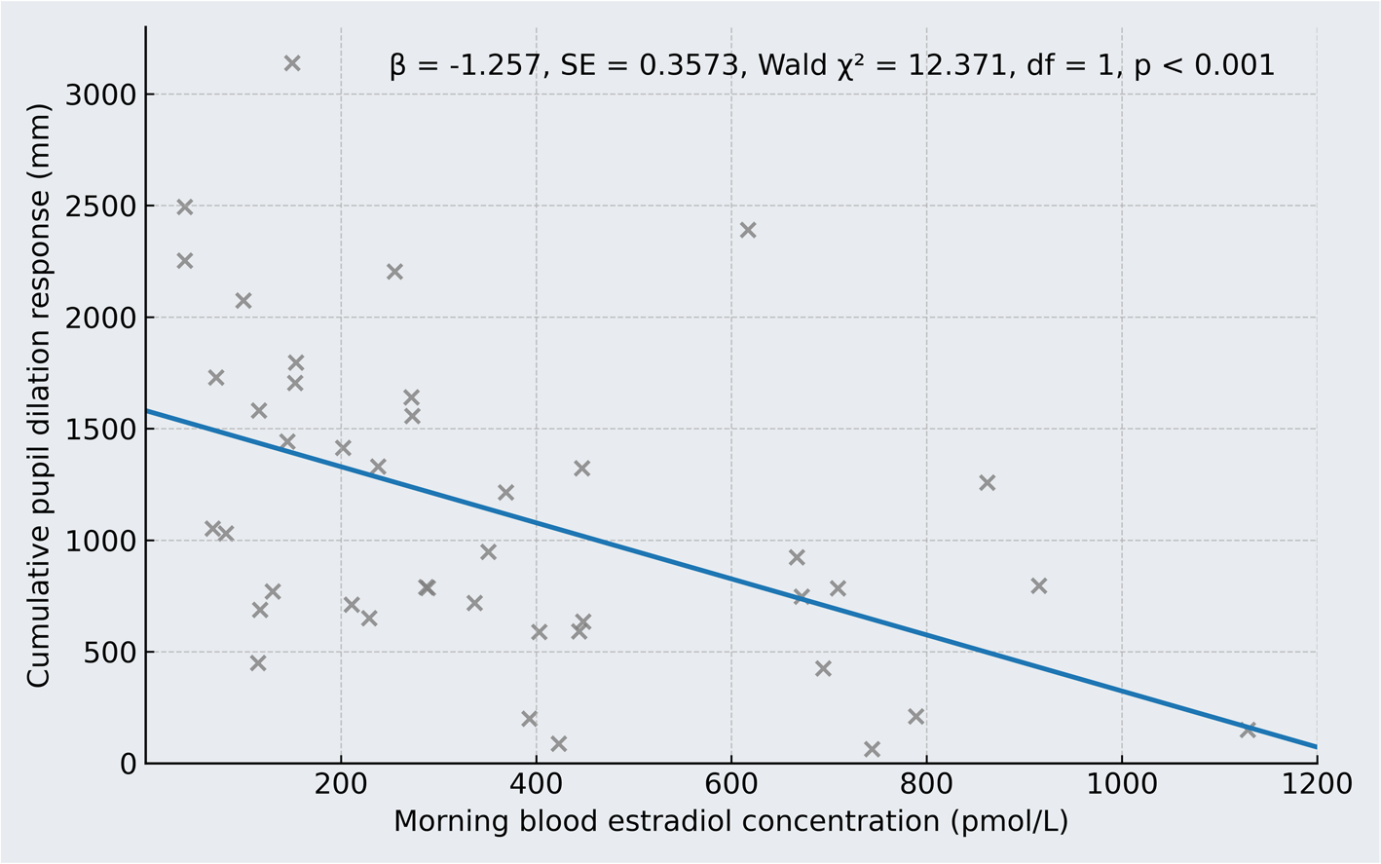
Elevated morning estradiol concentrations were significantly associated with a reduced cumulative peak pupil response during the karaoke replay. The blue line represents the predicted association, and the estimates shown are derived from a generalized linear model, with morning blood estradiol concentrations measured after sleep entered as a continuous predictor and cumulative pupil dilation as the outcome variable. Each gray “x” represents an individual participant’s observed data point. For adjusted estimates, see the Results section

When cumulative pupil dilation was used as the outcome variable (i.e., the sum of pupil diameters during the karaoke replay that exceeded the induvial upper bound of the 95% CI for baseline; group mean = + 1127 mm, range = + 64 to + 3139 mm), a significant negative association with estradiol levels was again observed. Women with higher morning estradiol concentrations exhibited a less pronounced cumulative pupil dilation response during the karaoke playback than those with lower levels (*B* = − 1.403 mm per change in pmol/L estradiol, SE = 0.3358, Wald χ^2^ = 17.454, df = 1, *p* < 0.001; Fig. 3 for the unadjusted association). Based on model estimates, for example, a 500 pmol/L difference in estradiol between two participants would correspond to an estimated 702 mm difference in cumulative pupil dilation—equivalent to a 0.26 mm difference in average pupil dilation above the baseline upper 95% CI during the karaoke playback. Indicative of a potential association between higher estradiol levels and more rapid post-stimulus recovery of pupil size, the inverse relationship between estradiol concentration and cumulative pupil dilation (group mean = + 382 mm, range = + 0.05 to + 1384 mm) persisted when the analysis was restricted to the post-karaoke period (*B* = − 0.617 mm per change in pmol/L estradiol, SE = 0.202, Wald χ^2^ = 9.355, df = 1, *p* = 0.002).

In contrast to estradiol, time spent in non-REM and REM sleep the night prior to the eye-tracking karaoke paradigm was not significantly associated with baseline-adjusted peak pupil diameters (non-REM: *B* = − 0.002 mm per change in minute non-REM, SE = 0.0017, Wald χ^2^ = 1.581, df = 1, *p* = 0.209; REM: *B* = 0.002 mm per change in minute REM, SE = 0.0018, Wald χ^2^ = 0.956, df = 1, *p* = 0.328). Given that non-REM sleep can be further divided into stages N1 (range = 10–70 min), N2 (range = 154–263 min), and N3 (range = 27–172 min), we ran a separate generalized linear model with time (min) in each stage as continuous predictors and adjusted peak pupil diameter as the outcome. No significant associations with peak pupil dilation during the karaoke replay were found (all *p* ≥ 0.334).

Similar to what we observed for peak pupil dilation, time spent in non-REM or REM sleep the night prior did not significantly predict cumulative pupil dilation, neither during the karaoke interval (non-REM: *B* = − 5.752 mm per change in minute non-REM, SE = 3.545, Wald χ^2^ = 2.633, df = 1, *p* = 0.105; REM: *B* = 6.101 mm per change in minute REM, SE = 3.853, Wald χ^2^ = 2.507, df = 1, *p* = 0.113) or during the post-karaoke interval (non-REM: *B* = − 1.578 mm per change in minute non-REM, SE = 2.129, Wald χ^2^ = 0.549, df = 1, *p* = 0.459; REM: *B* = 2.885 mm per change in minute REM, SE = 2.314, Wald χ^2^ = 1.555, df = 1, *p* = 0.212). When modeling time spent in each non-REM sleep stage separately, no significant associations with cumulative pupil dilation during the karaoke replay or post-karaoke intervals were found (all *p* ≥ 0.221).

### Subjective stress ratings

**Fig. 4:**
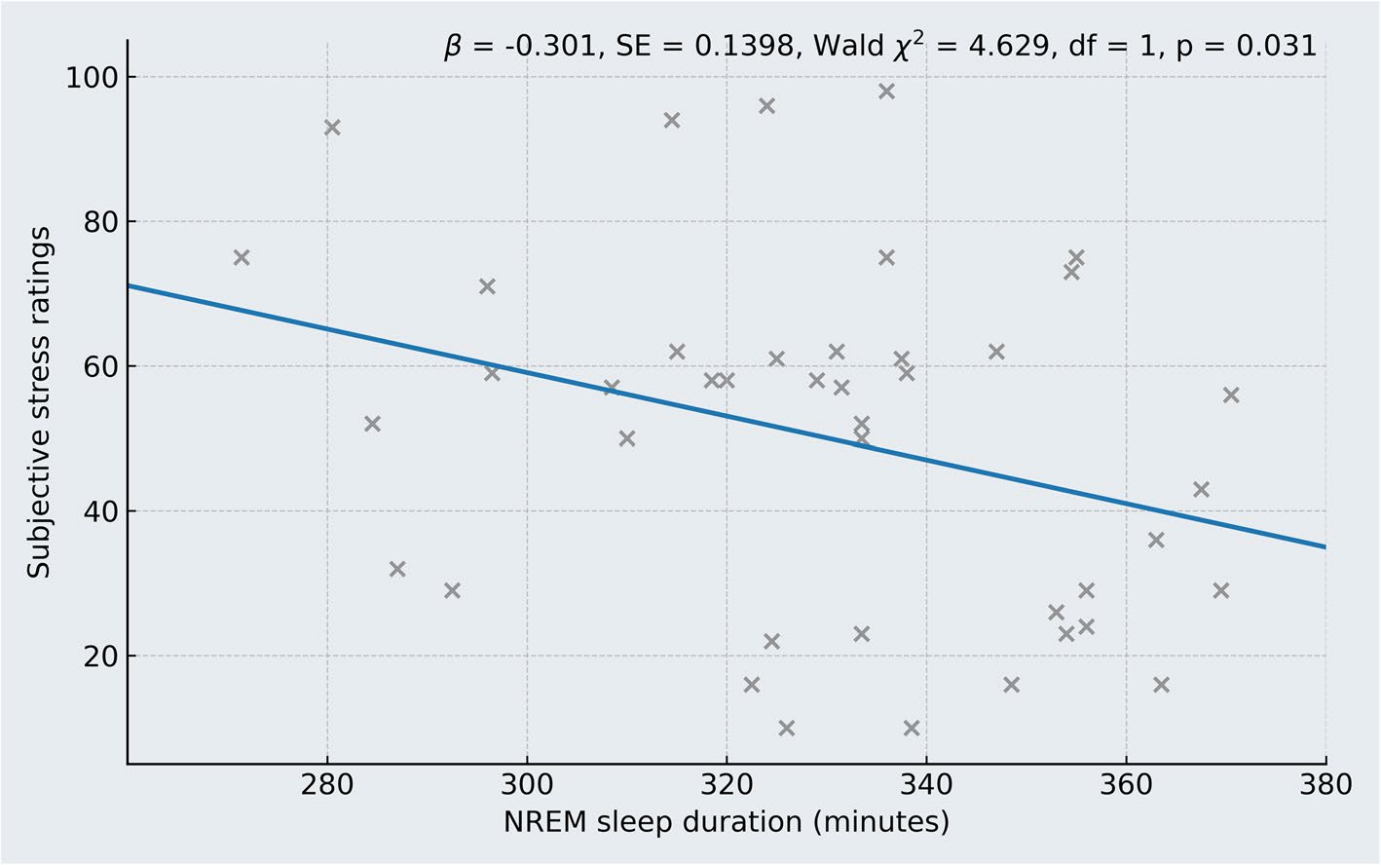
More time spent in non-REM sleep during the night was significantly associated with lower subjective stress during the karaoke task. The blue line represents the predicted association, and the estimates shown are derived from a generalized linear model, with time spent in non-REM sleep on the pre-test night entered as a continuous predictor and subjective stress ratings (range: 0–100 mm) as the outcome variable. Each gray “x” represents an individual participant’s observed data point. For adjusted estimates, see the Results section

As indicated by the post-karaoke 100-mm VAS (group mean = 50.2 mm, range = 10 to 98 mm), women felt less stressed by listening to the karaoke playback the more time they had spent in non-REM sleep the night before (*B* = − 0.313 mm per change in minute non-REM, SE = 0.1398, Wald χ^2^ = 5.016, df = 1, *p* = 0.025; Fig. 4 for the unadjusted association). In other words, a 60-min difference in non-REM sleep between two women would correspond to an estimated − 19 mm difference in their stress ratings. In contrast, neither time spent in REM sleep (*B* = − 0.059 mm per change in minute REM, SE = 0.1519, Wald χ^2^ = 0.151, df = 1, *p* = 0.697) nor morning blood estradiol levels (*B* = − 0.008 mm per change in pmol/L estradiol, SE = 0.0132, Wald χ^2^ = 0.362, df = 1, *p* = 0.547) significantly explained variance in subjective stress VAS scores.

When modeling the time spent in each non-REM sleep stage separately, the time in N3 was inversely associated with post-karaoke VAS scores for subjective stress (*B* = − 0.335 mm per change in minute N3, SE = 0.1564, Wald χ^2^ = 4.586, df = 1, *p* = 0.032). In contrast, time in N1 or N2 did not reach significance (*p* ≥ 0.138).

### Interaction analyses

None of the modeled interaction terms between blood levels of estradiol and time spent in non-REM or REM sleep was significantly associated with the outcomes of interest (*p* ≥ 0.214 for peak pupil dilation; *p* ≥ 0.512 for cumulative pupil dilation response during the karaoke replay; and *p* ≥ 0.402 for subjective stress ratings).

### Correlational analyses

Neither the peak pupil diameter nor the cumulative pupil dilation response during the karaoke phase was associated with subjective stress ratings assessed after karaoke playback (Pearson’s *r* for peak pupil dilation vs. subjective stress = − 0.055, *p* = 0.731; Pearson’s *r* for cumulative pupil dilation response vs. subjective stress = 0.087, *p* = 0.582). Additionally, blood estradiol concentrations were not significantly correlated with time in non-REM (Spearman’s *ρ* = 0.001, *p* = 0.993) or REM (Spearman’s *ρ* = 0.129, *p* = 0.417) sleep.

### Sensitivity analyses

Progesterone showed a strong floor effect in our sample, with 71.4% of participants having concentrations below 5.8 nmol/L. To address this, progesterone was dichotomized at 5.8 nmol/L based on the clinical reference [34], and the GzLMs were re-run including binary progesterone status as an additional exposure. Across all outcomes, blood estradiol concentrations remained significant, whereas progesterone did not.

For peak pupil dilation (peak dilation minus the individual’s upper baseline), estradiol concentrations were robustly associated (*B* = − 0.001, SE = 0.0002, Wald χ^2^ = 16.062, df = 1, *p* < 0.001), while progesterone status (luteal vs. non-luteal) did not differ significantly (parameter estimate (SE): + 1.09 (0.10) vs. + 0.88 (0.05), *p* = 0.098). For cumulative pupil dilation, estradiol concentrations again remained significant (*B* = − 1.824, SE = 0.439, Wald χ^2^ = 17.250, df = 1, *p* < 0.001), whereas progesterone status was not (luteal vs. non-luteal, parameter estimate (SE): 1397 (206) vs. 1020 (114) mm, *p* = 0.149). No significant estradiol × progesterone interaction was observed (peak: *p* = 0.855; cumulative: *p* = 0.655). When subjective stress ratings were used as the outcome, the GzLMs showed no significant associations with either estradiol (*B* = 0.002 mm per change in pmol/L estradiol, SE = 0.018, Wald χ^2^ = 0.014, df = 1, *p* = 0.907) or progesterone status (luteal vs. non-luteal, parameter estimate (SE): 43.8 (8.2) vs. 52.8 (4.6) mm, *p* = 0.392). No estradiol × progesterone interaction was found (*p* = 0.138).

A separate set of sensitivity analyses, in which participants’ emotional stability scores were removed from the GzLMs, showed that blood concentrations of estradiol remained a robust predictor of both peak (*B* = − 0.001, SE = 0.0002, Wald χ^2^ = 12.630, df = 1, *p* < 0.001) and cumulative pupil responses (*B* = − 1.382, SE = 0.347, Wald χ^2^ = 15.850, df = 1, *p* < 0.001), whereas non-REM and REM durations were not significantly associated with these outcomes (all *p* ≥ 0.120). Furthermore, for subjective stress ratings, greater non-REM duration continued to predict lower ratings (*B* = − 0.306, SE = 0.139, Wald χ^2^ = 4.844, df = 1, *p* = 0.028), while estradiol (*p* = 0.558) and REM duration (*p* = 0.702) remained nonsignificant predictors.

Finally, when TST was used as the sole sleep predictor in our GzLMs, it was not associated with peak pupil dilation (*B* = 0.000, SE = 0.0013, Wald χ^2^ = 0.065, df = 1, *p* = 0.798) or cumulative pupil responses (*B* = − 0.335, SE = 2.815, Wald χ^2^ = 0.014, df = 1, *p* = 0.905). For subjective stress ratings, the association was in the same direction as N3 sleep (see above), but it did not reach conventional significance (*B* = − 0.197, SE = 0.1065, Wald χ^2^ = 3.424, df = 1, *p* = 0.064).

## Discussion

The present study aimed to examine whether inter-individual differences in estradiol levels and sleep architecture predict variability in responses to social-evaluative stress among women. To this end, we assessed subjective stress ratings and pupil dilation, a well-established physiological index of SNS activation [23, 24], in 42 healthy, naturally cycling young women. This sample size falls at the upper end of the range commonly used in previous experimental studies on sleep and emotional processing [11–15].

Our results showed that women with higher pre-stress estradiol levels exhibited significantly lower autonomic activation, as indexed by reduced pupil dilation during the karaoke playback, a socially evaluative task. This suggests that elevated estradiol may attenuate autonomic arousal in response to social-evaluative stress. This interpretation is supported by animal studies in which 17β-estradiol administration in ovariectomized rats acutely reduced renal and splanchnic nerve activity and heart rate without affecting mean arterial pressure, indicating a central sympatho-inhibitory effect [36]. Moreover, estradiol has been shown to dampen activity in the medial amygdala [37], a brain region critical for initiating autonomic responses to threat [38].

It is important to note, however, that not all studies in women support a consistently protective role of estradiol in psychological stress resilience [18, 19]. Beyond estradiol, progesterone has been implicated in modulating mood, emotion processing [39], sleep [40, 41], and stress sensitivity [42]. In the present study, progesterone levels showed a strong floor effect, with approximately 70% of participants displaying concentrations below 5.8 nmol/L, consistent with values typically observed outside the luteal phase [34, 35]. To address this, we conducted sensitivity analyses considering progesterone status (luteal vs. non-luteal) as an additional exposure. Estradiol remained significantly associated with pupil responses, whereas progesterone did not, and the associations between estradiol and outcome measures did not vary by progesterone status. These findings suggest that inter-individual differences in estradiol may shape the autonomic response to social-evaluative stress. Further studies are warranted to determine whether intra-individual fluctuations in estradiol similarly influence autonomic arousal to stressors like the one applied in the present study.

A second key finding was that greater time spent in non-REM stage N3 sleep the night before testing was associated with lower subjective stress ratings following the karaoke task. However, N3 sleep duration did not correlate with pupil dilation. This dissociation suggests that subjective stress appraisal and autonomic arousal in response to social-evaluative stress may be governed by distinct underlying mechanisms. Pupil dilation reflects rapid, automatic activation of the SNS [23, 24], which may not necessarily result in the conscious feeling of stress, as it is a physiological response that occurs outside of conscious awareness [43]. In contrast, subjective stress ratings rely on cognitive appraisal, a higher-order process involving conscious evaluation, emotional interpretation, and the attribution of meaning to the stressor [43]. This distinction may also help explain why pupil dilation and post-task stress ratings were not correlated. Furthermore, the finding that estradiol was associated with the autonomic response to the social-evaluative stress task, while time spent in non-REM sleep (particularly stage N3) was linked to subjective stress appraisal, may help explain the absence of an interaction between estradiol and sleep in psychological stress processing.

Why might N3 sleep selectively influence cognitive but not autonomic stress responses? One plausible explanation is that prior research has shown that N3-rich sleep enhances functional connectivity between the prefrontal cortex and amygdala, and this connectivity is associated with reduced anxiety the following day [13]. The prefrontal cortex is involved in cognitive appraisal processes, including assessing threat magnitude, controllability, and available coping strategies [44]. While autonomic responses, such as pupil dilation, are immediate and automatic, cognitive processes like emotional regulation and psychological stress appraisal depend on higher-order brain functions. Thus, more time in N3 sleep may help buffer individuals from heightened subjective emotional responses to social stress by preserving prefrontal cortex-mediated evaluation.

Interestingly, time spent in REM sleep the night before the stressor was not associated with either subjective stress ratings or pupil dilation responses. This may suggest that REM sleep plays a more crucial role in the overnight processing and resolution of past emotional experiences [10]. For example, a study using a similar karaoke task with participants from the Netherlands Sleep Registry found that amygdala reactivity to playback of participants’ own singing decreased after sleep, but only among those with normal sleep patterns. In contrast, individuals with insomnia and frequent REM sleep disruptions showed the least reduction in amygdala response [45], suggesting impaired emotional depotentiation during REM sleep.

## Limitations

Several limitations should be acknowledged. As sleep was not experimentally manipulated in the present study (e.g., through selective sleep stage suppression protocols), no firm conclusions can be drawn about how REM and non-REM sleep durations outside the investigated time window may influence next-day autonomic and cognitive responses to psychological stressors. Another limitation concerns the stress paradigm used. Although the karaoke replay task offers high ecological validity by modeling unanticipated social evaluation, it may not elicit stress responses as robustly as more established paradigms, such as the Trier Social Stress Test [46]. However, the present singing paradigm shares critical features with a similar singing-based procedure that has been shown to evoke autonomic responses comparable to those induced by the Trier Social Stress Test [30]. Importantly, none of our participants was a professional singer or had extensive public performance experience, reducing the likelihood of desensitization or familiarity effects, yet it cannot be ruled out that previous karaoke experience may have influenced their responses to the task. Repeated measures of subjective stress after the cessation of the stress paradigm, including assessment of other emotions such as embarrassment, would have provided a better understanding of the duration of social-evaluative stress effects; therefore, future studies should include multiple post-stressor assessments to capture the temporal dynamics of subjective stress responses. Finally, the associations reported in this study are correlational and should not be interpreted as evidence of causality. Further interventional research, such as studies conducted at different phases of the menstrual cycle, or those involving oral contraceptives or selective sleep disruption, is needed to establish causal links.

## Conclusions

Our findings demonstrate that estradiol and non-REM sleep independently shape distinct aspects of the human stress response. Higher pre-stress estradiol levels were linked to reduced autonomic arousal, while longer non-REM sleep duration was associated with lower subjective stress following a socially evaluative challenge. These results suggest that hormonal and sleep-related factors act through separate but complementary pathways, one dampening physiological arousal, the other modulating stress appraisal. Importantly, these biological processes may contribute to individual differences in vulnerability or resilience to stress-related mental health conditions. By integrating endocrine and sleep physiology, this study advances a more comprehensive understanding of the mechanisms through which the body and brain respond to psychosocial threat, with implications for early identification and prevention of stress-related disorders in women.

## Data Availability

Data presented herein are available as deidentified datasets upon request to researchers at other institutions, contingent upon signing a data access agreement prior to release. For inquiries, please contact Pei Xue (pei.xue@uu.se).

## Abbreviations

ANS: Autonomic nervous system
EEG: Electroencephalography
FSH: Follicle-stimulating hormone
GzLMs: Generalized linear models
LH: Luteinizing hormone
N3: Non-REM stage 3
REM: Rapid eye movement
SNS: Sympathetic nervous system
TST: Total sleep time
VAS: Visual analog scale
95% CI: 95% Confidence interval
